# An amplitude-based characteristic parameter extraction algorithm for cerebral edema detection based on electromagnetic induction

**DOI:** 10.1186/s12938-021-00913-4

**Published:** 2021-08-03

**Authors:** Jingbo Chen, Gen Li, Huayou Liang, Shuanglin Zhao, Jian Sun, Mingxin Qin

**Affiliations:** 1 College of Biomedical Engineering, Third Military Medical University (Army Medical University), Chongqing, China; 2 School of Pharmacy and Bioengineering, Chongqing University of Technology, Chongqing, China; 3 China Aerodynamics Research and Development Center Low Speed Aerodynamic Institute, Mianyang, Sichuan China

**Keywords:** Cerebral edema, Electromagnetic induction, Ab-CPE algorithm, Multi-frequency characteristic analysis

## Abstract

**Background:**

Cerebral edema is a common condition secondary to any type of neurological injury. The early diagnosis and monitoring of cerebral edema is of great importance to improve the prognosis. In this article, a flexible conformal electromagnetic two-coil sensor was employed as the electromagnetic induction sensor, associated with a vector network analyzer (VNA) for signal generation and receiving. Measurement of amplitude data over the frequency range of 1–100 MHz is conducted to evaluate the changes in cerebral edema. We proposed an Amplitude-based Characteristic Parameter Extraction (Ab-CPE) algorithm for multi-frequency characteristic analysis over the frequency range of 1–100 MHz and investigated its performance in electromagnetic induction-based cerebral edema detection and distinction of its acute/chronic phase. Fourteen rabbits were enrolled to establish cerebral edema model and the 24 h real-time monitoring experiments were carried out for algorithm verification.

**Results:**

The proposed Ab-CPE algorithm was able to detect cerebral edema with a sensitivity of 94.1% and specificity of 95.4%. Also, in the early stage, it can detect cerebral edema with a sensitivity of 85.0% and specificity of 87.5%. Moreover, the Ab-CPE algorithm was able to distinguish between acute and chronic phase of cerebral edema with a sensitivity of 85.0% and specificity of 91.0%.

**Conclusion:**

The proposed Ab-CPE algorithm is suitable for multi-frequency characteristic analysis. Combined with this algorithm, the electromagnetic induction method has an excellent performance on the detection and monitoring of cerebral edema.

## Background

Cerebral edema, which can be defined as the abnormal increase and accumulation of intracranial fluid, is a common condition secondary to stroke and traumatic brain injury (TBI). Cerebral edema has been associated with high morbidity, high mortality, and high disability [1–4]. Pathologically, cerebral edema can be divided into two subtypes: cytotoxic and vasogenic cerebral edema. In the early stage of cerebral edema, it is mainly cytotoxic edema characterized by intracellular accumulation of fluid and Na^+^ resulting in cell swelling. Several hours after the onset of cerebral edema, intracranial changes gradually become dominated by vasogenic cerebral edema. At this stage, it is characterized by extracellular accumulation of fluid resulting from disruption of the blood–brain barrier (BBB) and extravasations of serum proteins [2]. It has been reported that effective monitoring methods and timely intervention can help improve the prognosis [5–7]. Effective cerebral edema monitoring is still necessary which will help medical staff to adjust the therapeutic schedule in time.

The current gold standard for the diagnosis of cerebral edema is mainly based on imaging methods, such as Computed Tomography (CT) and Magnetic Resonance Imaging (MRI) [8]. The cost of CT or MRI scan is relatively expensive and these devices require expert operators. Also, CT scans are based on radiation, so there is difficulty in performing repeat scans on children. On the other hand, most imaging equipment is usually located in the radiology department. Thus, patients need extra referral for imaging, which increases the difficulty and procedures of nursing, especially for severe cases. In addition, early diagnosis requires collection of patient's physiological information as soon as possible. However, it is difficult for patients to undergo CT or MRI examinations in the first place. Besides, the imaging equipment is large and fit, which means that the traditional imaging methods cannot carry out bedside monitoring. The most widely used bedside monitoring tool is Intracranial Pressure (ICP) monitoring [9]. According to different measurement needs, the ICP probe can be inserted into the subdural, cerebral ventricle, and parenchyma [10]. However, as an invasive method, ICP causes secondary injury to patients, which may adversely affect the prognosis. In addition, ICP itself has not been currently an internationally recognized indicator of bedside monitoring [11]. Recent research found that ICP monitoring could hardly improve the functional outcome of moderate TBI, although it may possibly reduce the in-hospital mortality [12]. Another research also found that there was no relationship between ICP monitoring and clinical outcome for patients with Glasgow Coma Scale (GCS) scores of 3–8 [9].

In view of this situation, non-invasive bedside diagnosis and monitoring methods have become an attractive research field in recent years [13, 14]. Transcranial Doppler (TCD) utilizes ultrasound to detect variations in cerebra blood flow velocity of specific intracranial blood vessels (such as the middle artery), through which it can assess the level of intracranial blood supply [15]. However, TCD highly depends on medical staff and requires professional training. Generally, it can only be done by an ultrasound technician. Near-infrared spectroscopy (NIR) can be used as a monitoring tool of cerebral and myocardial oxygenation [16]. However, the measurement depth of NIR is limited [17]. Electrical Impedance Tomography (EIT) method measures the dielectric properties of biological tissues. Amid that different biological tissues have different electrical conductivity and permittivity at specific frequencies, lesions such as stroke or TBI which causes changes in intracranial components lead to changes in brain’s dielectric parameters. However, EIT requires multiple electrodes attached to the scalp. The contact impedance brings inevitable interference to the measurement results [18]. Also, the skull, as a high resistance layer, will block the passage of current, which weakens the EIT signal [19]. The electromagnetic induction detection method is also based on the dielectric properties of biological tissues [20]. The advantage of this method is that the skull does not act as a barrier for the passage of electromagnetic waves [21]. This method uses electromagnetic waves to pass through the brain. Under certain pathological conditions (such as cerebral hemorrhage, cerebral ischemia, cerebral edema, etc.), the change of brain’s dielectric properties can be measured via the signal from the receiving antenna [22]. Electromagnetic induction detection has developed many research branches. For example, Capacitively Coupled Electrical Impedance Tomography (CCEIT) uses low-frequency electric field to study the permittivity of biological tissues [19, 23]. However, the capacitive sensor used in the CCEIT method has low sensitivity in the deep part of the brain. There are also studies focusing on microwave-based detection, including microwave-based diagnosis [24, 25] and microwave imaging that use multiple antennas for discrimination [26–29]. In addition, there are studies utilizing low-frequency magnetic induction method to measure vital signs [30–33], intracranial lesions [34–36], cancer [37–39], osteoporosis [40], suit fit [41], etc.

Electromagnetic induction has high potential application prospects in the field of diagnosis and monitoring of specific brain diseases. Oziel et al. utilized the Z-parameter to monitor the accumulation of blood in the head [42]. Teichmann et al. proposed a non-contact magnetic induction monitoring device that can measure pulse and respiratory activity via variation in the resonance frequency of the oscillatory circuitry [43–46]. Griffith developed a skin patch sensor and measure intracranial fluid-volume change via S-parameters from 700 MHz to 1.1 GHz [20]. Jiang et al. used an open-ended cylindrical waveguide for continuous assessment of intracerebral hemorrhage utilizing the $S_{11}$ parameter in a range of 100–400 MHz [29]. Saied et al. designed an antenna system that propagates at 800 MHz and 2.1 GHz, respectively for neurodegeneration monitoring. The resonant nature of the sensor, which can be assessed by $S_{11}$, can be used to differentiate between detecting brain atrophy and lateral ventricle enlargement [47]. However, there still are some unsolved issues in existing studies. Previous measurement methods, which only relies on the change trend of the phase shift of a single frequency or the frequency shift of the characteristic frequency, has been proved that it cannot be directly applied to detection and monitoring due to the complexity of the physiological structure and regulation mechanism of brain. There were studies showing that brain cannot be simply equivalent to a multilayer dielectric structure [42, 48, 49]. Pathological changes inside the brain cause one or more variations including intracranial volume, conductivity, permittivity, and the relative proportions of intracranial tissues. It is difficult to make diagnosis only based on the phase shift or frequency shift data. More importantly, this change is not linear. Our recent animal research based on the epidural freezing edema model has found that the electromagnetic induction signal and ICP showed fast change in the first 6 h on account of the strong intracranial compensatory effect. The change rate gradually decreased from 6 to 24th h with the exhaustion of compensatory [50]. According to the change trend of ICP and electromagnetic induction signals, it can be considered that in this rabbit edema model, the first 6 h is the acute phase of cerebral edema and the latter 6–24 h is the chronic phase. If the magnetic induction signals can provide hints at these two different stages of cerebral edema, this method may become a new indicator for early intervention. On the other hand, the latest research by Oziel et al. also showed that the amplitude/phase change has an increase or decrease or even a non-linear trend with the injected blood volume at different measurement frequencies [51–54]. At present, some studies have begun to use multi-frequency data combined with specific algorithms. Gen Li used multi-frequency reflection and transmission characteristics, combined with BP algorithm, to carry out brain edema monitoring [55]. Oziel also presented a single coil inductive device and the attendant algorithm for detection of changes in fluid/tissue ratio [52]. These studies were all targeted to use algorithms for *S*-parameter characteristic extraction and further analysis. Consequently, using proper algorithms to extract multiple parameters and multi-dimensional information can improve detection capabilities. Based on the above studies, this article proposes a hypothesis that the electromagnetic induction-based detection and distinction of acute/chronic phase of cerebral edema can be realized through a specialized characteristic parameter extraction algorithm.

In this study, an Amplitude-based Characteristic Parameter Extraction (Ab-CPE) algorithm was proposed based on the principle of multi-frequency electromagnetic induction and two-port network theory. For characteristic analysis, a 24-h monitoring experiment was carried out utilizing the rabbit liquid nitrogen freezing brain edema model. In particular, the performance of brain edema detection and the distinction of acute/chronic phase was investigated. This research is expected to provide algorithmic foundation for the clinical application of electromagnetic induction methods and bedside monitoring of cerebral edema.

## Results

### Results of $S_{21}$ amplitude–frequency curve

**Fig. 1:**
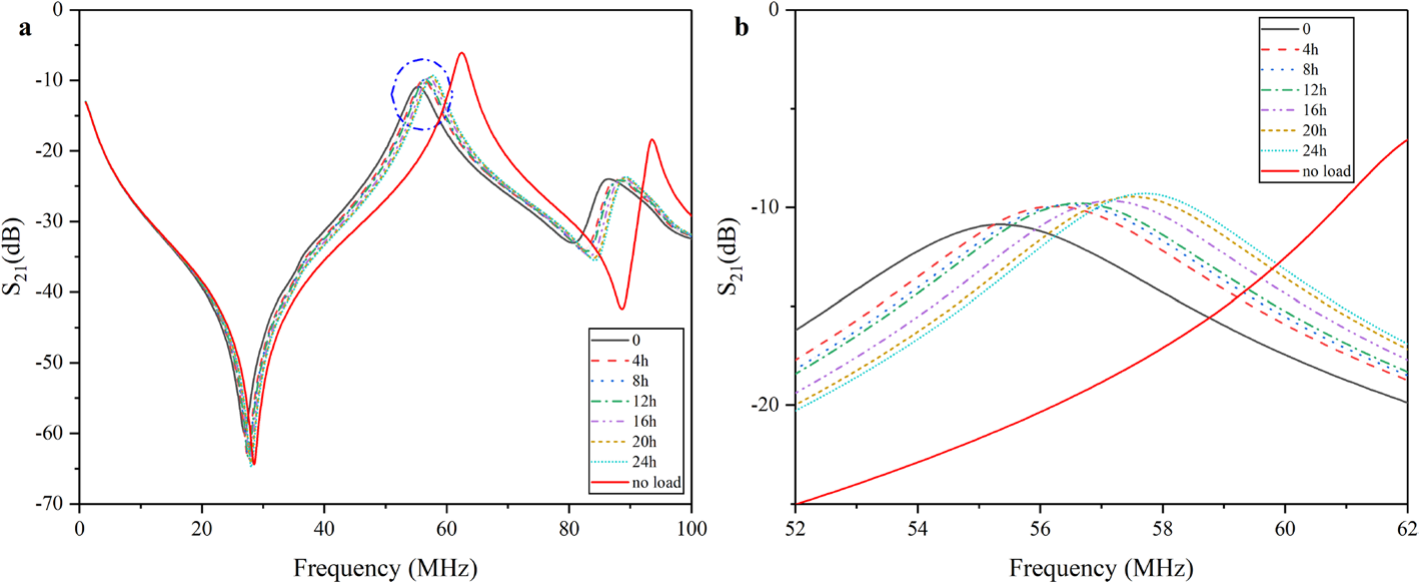
Amplitude–frequency curve of rabbit Exp.6 in 24 h. **a** Measurement data in 1–100 MHz. **b** Measurement data near characteristic frequency (52–62 MHz)

For illustration, Fig. 1 shows the amplitude-frequency data of rabbit Exp.6 in the experimental group at 4 h interval. It can be seen from Fig. 1a that when rabbit's brain is placed in the two-port network, its dielectric properties will affect the transmission parameters, resulting in various amplitude of $S_{21}$ at each frequency in 1–100 MHz, which is consistent with previous findings [20, 52]. The dashed line in Fig. 1a showed its characteristic frequency. For further observation, Fig. 1b draws the amplitude–frequency curve within 52–62 MHz. It can be found that there are multiple variation characteristics in this curve. First, the peak value gradually increases and the characteristic frequency also shows a frequency shift trend.

**Fig. 2:**
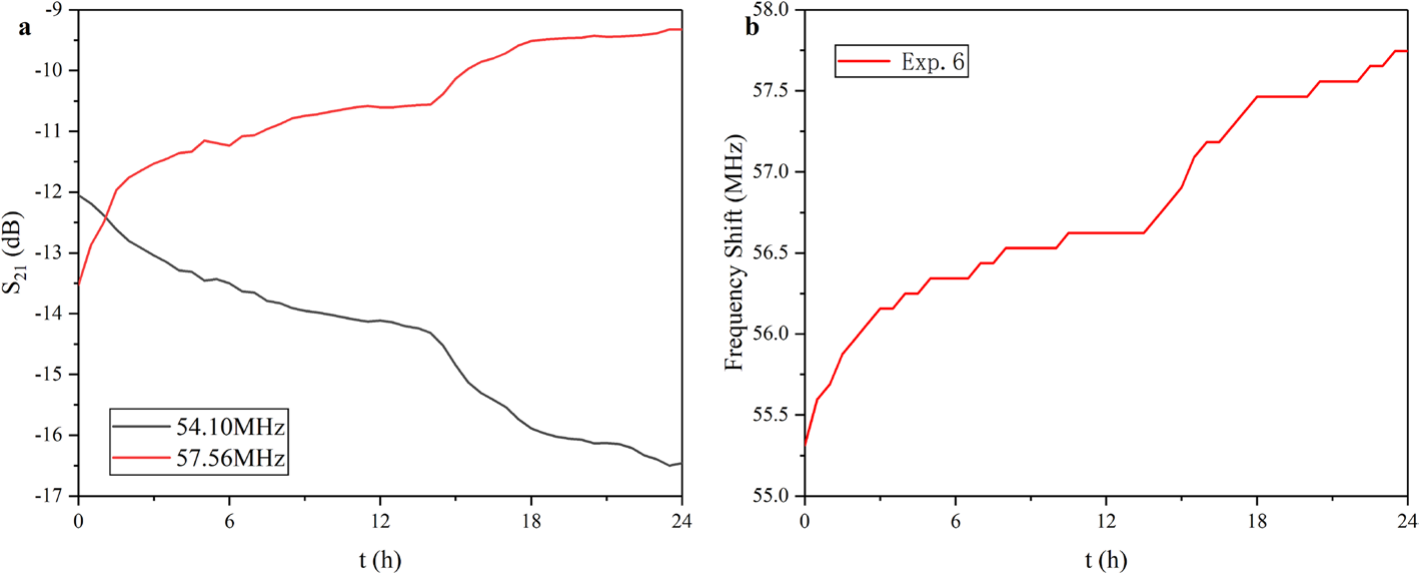
**a** Change trend of the $S_{21}$ in 54.1 MHz and 57.56 MHz as a function of time. **b** Frequency shift trend of characteristic frequency as a function of time

Next, two frequency points near the characteristic frequency in the amplitude–frequency curve were selected to observe the change trend of the amplitude over time (54.10 MHz, 57.56 MHz). Results are shown in Fig. 2a. It can be seen that $S_{21}$ at 54.1 MHz showed a downward trend. The decline rate was relatively linear within 0-15 h and gradually changed faster in 15–18 h. And finally, it tended to be stable again. At 57.56 MHz, $S_{21}$ showed an upward trend, in which it changed fast within 3 h, and gradually slowed down in subsequent hours. Although change rate also increased during the 15–18 h period, the trend was not as obvious as that at 54.1 MHz. Figure 2b plots the frequency shift trend of the characteristic frequency. It can be found that the characteristic frequency gradually increased. This trend was relatively obvious in the first 6 h and progressively stabilized in 6–15 h. Then, it started increasing again in 15–24 h. It can be concluded that $S_{21}$ contains rich variation characteristics near the characteristic frequency. Observing only the frequency shift or the amplitude change of $S_{21}$ at one frequency will miss part of the data trends [53].

### Detection of cerebral edema

**Fig. 3:**
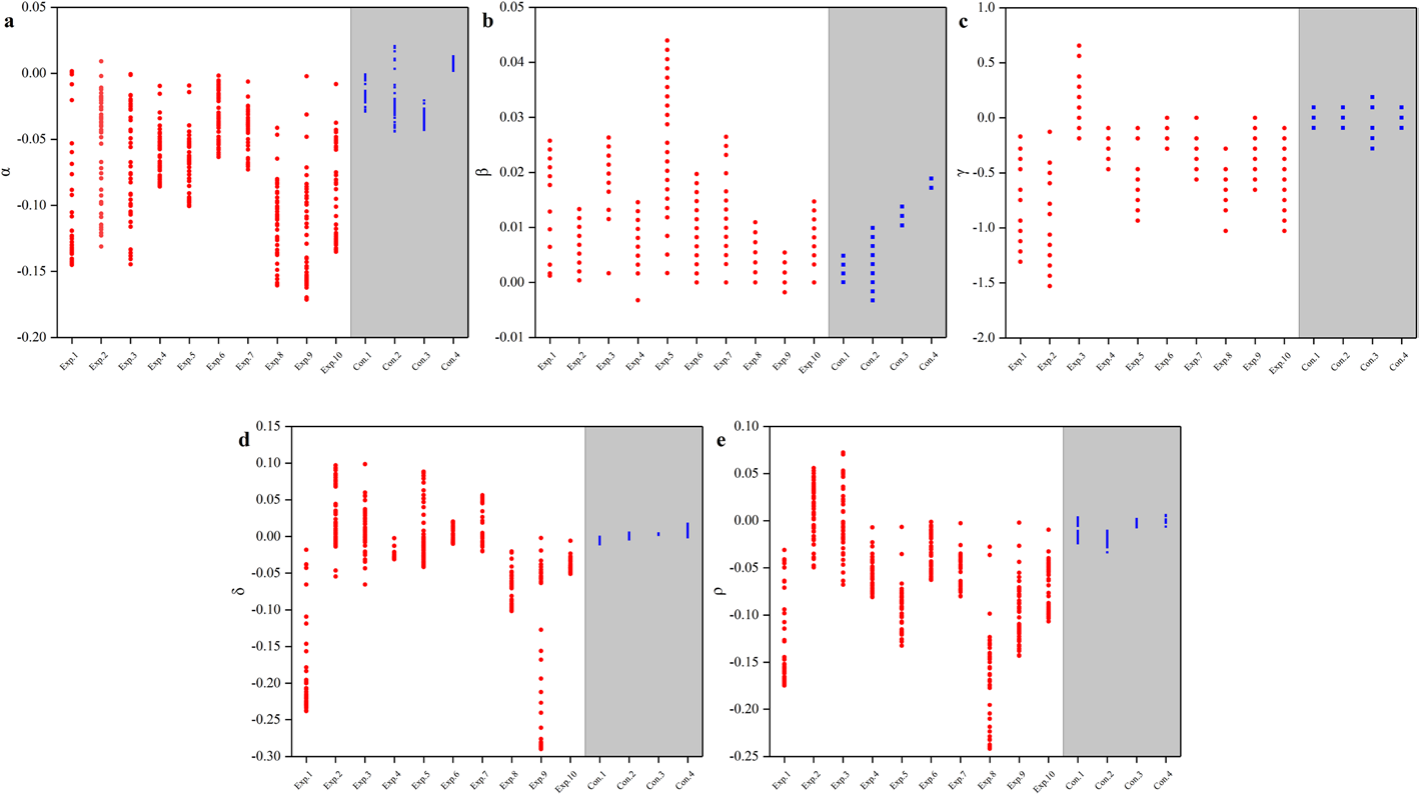
Characteristic parameters in experimental group and control group. Ten rabbits (Exp.1–Exp.10) in experimental group were listed with white background. Four rabbits (Con.1–Con.4) in control group were listed with gray background

Figure 3 shows the characteristic parameters in experimental group and control group obtained by the Ab-CPE algorithm. All the five characteristic parameters of the experimental group had clear changes. In contrast, the characteristic parameters of control group had little change, especially for $\delta$ and $\rho$.

Table 1 lists the Receiver-Operating Characteristic (ROC) data of experimental group vs control group based on every possible combination of characteristic parameters. ROC is evaluated by Area Under Curve (AUC), where AUC $\in \left[ {0,1} \right]$ and higher AUC represents better detection capability. The AUC of single characteristic parameters are relatively poor, of which all were lower than 0.9. Among those multi-parameter combinations, AUC of $\gamma \delta \rho$ was highest, reaching 0.9699.AUC of $\gamma \delta$ and $\gamma \rho$ is close to $\gamma \delta \rho$, reaching 0.9657 and 0.9658, respectively. Thus, $\gamma \delta \rho$ can be selected as the detection characteristic parameters of cerebral edema.

**Table 1:** ROC results of cerebral edema detection in 24 h based on every possible combination of characteristic parameters

Single		Double		Triple		Quadruple		Quintuple	
Characteristic parameter	AUC^*^	Characteristic parameter	AUC	Characteristic parameter	AUC	Characteristic parameter	AUC	Characteristic parameter	AUC
$\alpha$	0.89	$\alpha \beta$	0.84	$\alpha \beta \gamma$	0.90	$\alpha \beta \gamma \delta$	0.89	$\alpha \beta \gamma \delta \rho$	0.92
$\beta$	0.72	$\alpha \gamma$	0.92	$\alpha \beta \delta$	0.87	$\alpha \beta \gamma \rho$	0.95		
$\gamma$	0.87	$\alpha \delta$	0.90	$\alpha \beta \rho$	0.87	$\alpha \beta \delta \rho$	0.88		
$\delta$	0.64	$\alpha \rho$	0.90	$\alpha \gamma \delta$	0.94	$\alpha \gamma \delta \rho$	0.92		
$\rho$	0.74	$\beta \gamma$	0.84	$\alpha \gamma \rho$	0.95	$\beta \gamma \delta \rho$	0.91		
		$\beta \delta$	0.75	$\alpha \delta \rho$	0.91				
		$\beta \rho$	0.77	$\beta \gamma \delta$	0.88				
		$\gamma \delta$	0.97	$\beta \gamma \rho$	0.89				
		$\gamma \rho$	0.97	$\beta \delta \rho$	0.78				
		$\delta \rho$	0.94	$\gamma \delta \rho$	0.97				

*AUC takes two significant digits

**Fig. 4:**
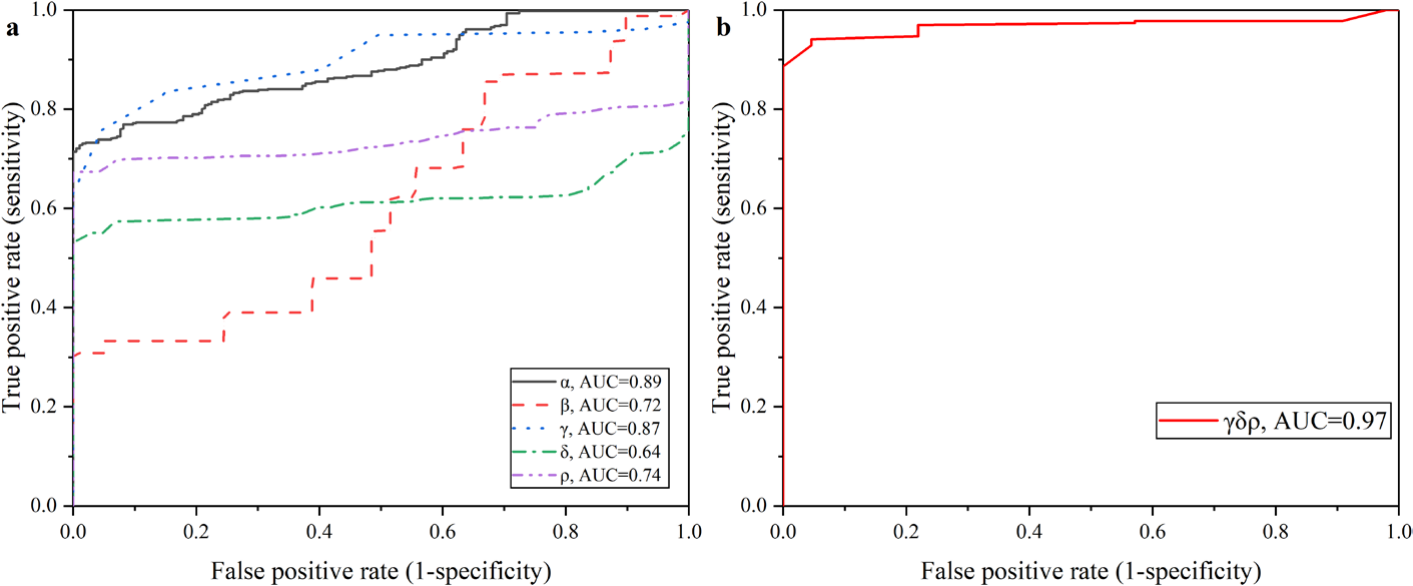
ROC curve of experimental group vs control group distinction within 24 h. **a** ROC curves based on single characteristic parameter; **b** ROC curves based on $\gamma \delta \rho$

Figure 4a, b plots the ROC curves based on single characteristic parameter and combination $\gamma \delta \rho$. The selected $\gamma \delta \rho$ parameters can achieve high sensitivity and specificity, reaching 94.1% and 95.4%, respectively.

At the same time, we also selected the data within 1 h to evaluate the performance of Ab-CPE algorithm in early detection of cerebral edema. Results are shown in Table 2. Consequently, the ROC result in early stage was slightly inferior to that in the whole 24 h, but the highest AUC, the combination of $\gamma \delta \rho$, still reached 0.88, which consisted with the results in Table 1.

**Table 2:** ROC results of cerebral edema detection in 1 h based on every possible combination of characteristic parameters

Single		Double		Triple		Quadruple		Quintuple	
Characteristic parameter	AUC^*^	Characteristic parameter	AUC	Characteristic parameter	AUC	Characteristic parameter	AUC	Characteristic parameter	AUC
$\alpha$	0.77	$\alpha \beta$	0.45	$\alpha \beta \gamma$	0.55	$\alpha \beta \gamma \delta$	0.64	$\alpha \beta \gamma \delta \rho$	0.67
$\beta$	0.71	$\alpha \gamma$	0.71	$\alpha \beta \delta$	0.51	$\alpha \beta \gamma \rho$	0.84		
$\gamma$	0.72	$\alpha \delta$	0.62	$\alpha \beta \rho$	0.53	$\alpha \beta \delta \rho$	0.59		
$\delta$	0.59	$\alpha \rho$	0.69	$\alpha \gamma \delta$	0.74	$\alpha \gamma \delta \rho$	0.62		
$\rho$	0.73	$\beta \gamma$	0.44	$\alpha \gamma \rho$	0.83	$\beta \gamma \delta \rho$	0.62		
		$\beta \delta$	0.61	$\alpha \delta \rho$	0.71				
		$\beta \rho$	0.56	$\beta \gamma \delta$	0.61				
		$\gamma \delta$	0.82	$\beta \gamma \rho$	0.56				
		$\gamma \rho$	0.87	$\beta \delta \rho$	0.65				
		$\delta \rho$	0.81	$\gamma \delta \rho$	0.88				

^*^AUC takes two significant digits

**Fig. 5:**
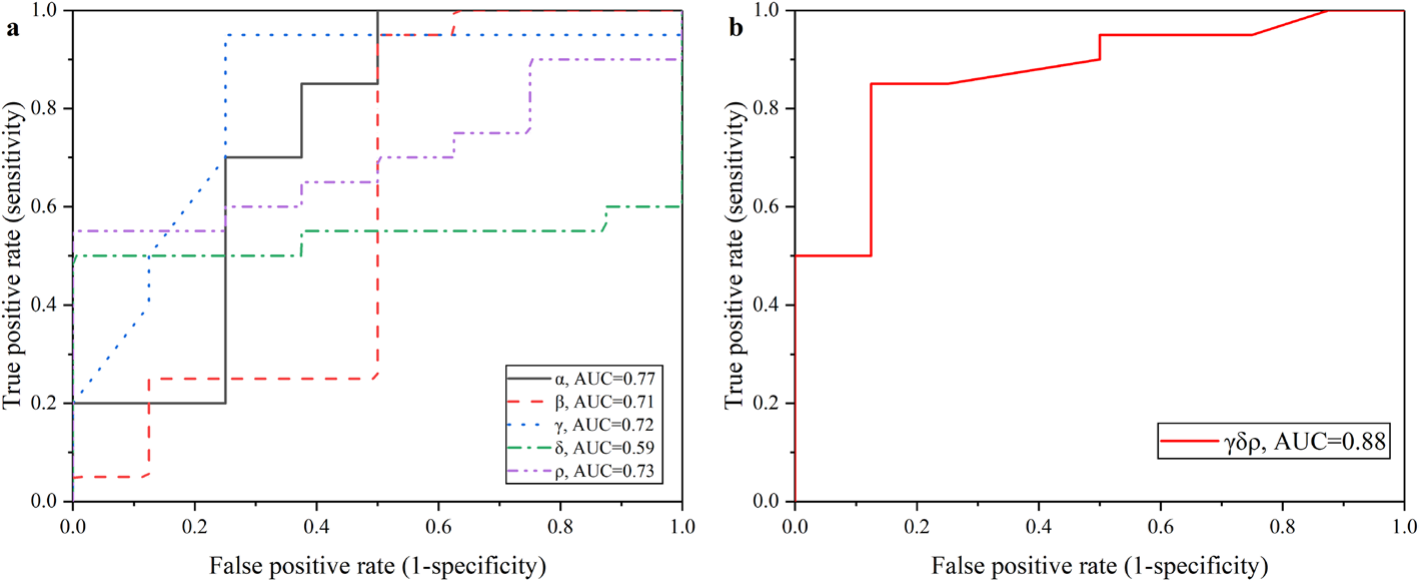
ROC curve of experimental group vs control group distinction within 1 h. **a** ROC curves based on single characteristic parameter; **b** ROC curves based on $\gamma \delta \rho$

Figure 5a, b plots the ROC curve within the first hour of experimental group vs control group. The optimal discrimination index based on $\gamma \delta \rho$ reached 85.0% sensitivity and 87.5% specificity. The performance of Ab-CPE algorithm-based detection had indeed weakened in the early stage. There may be errors caused by insufficient data. There are not many data sampling points within 1 h. In this experiment, data are sampled every 30 min, so there are only two rounds of data within 1 h that can be enrolled for analysis. In the next step, the sampling rate will be increased in early stage. In all, the combination of $\gamma \delta \rho$ has the highest AUC both in 24 h and within 1 h. It can be concluded that $\gamma \delta \rho$ is the optimal combination choice for the detection and early warning of cerebral edema.

### Distinction of acute/chronic phase of cerebral edema

**Fig. 6:**
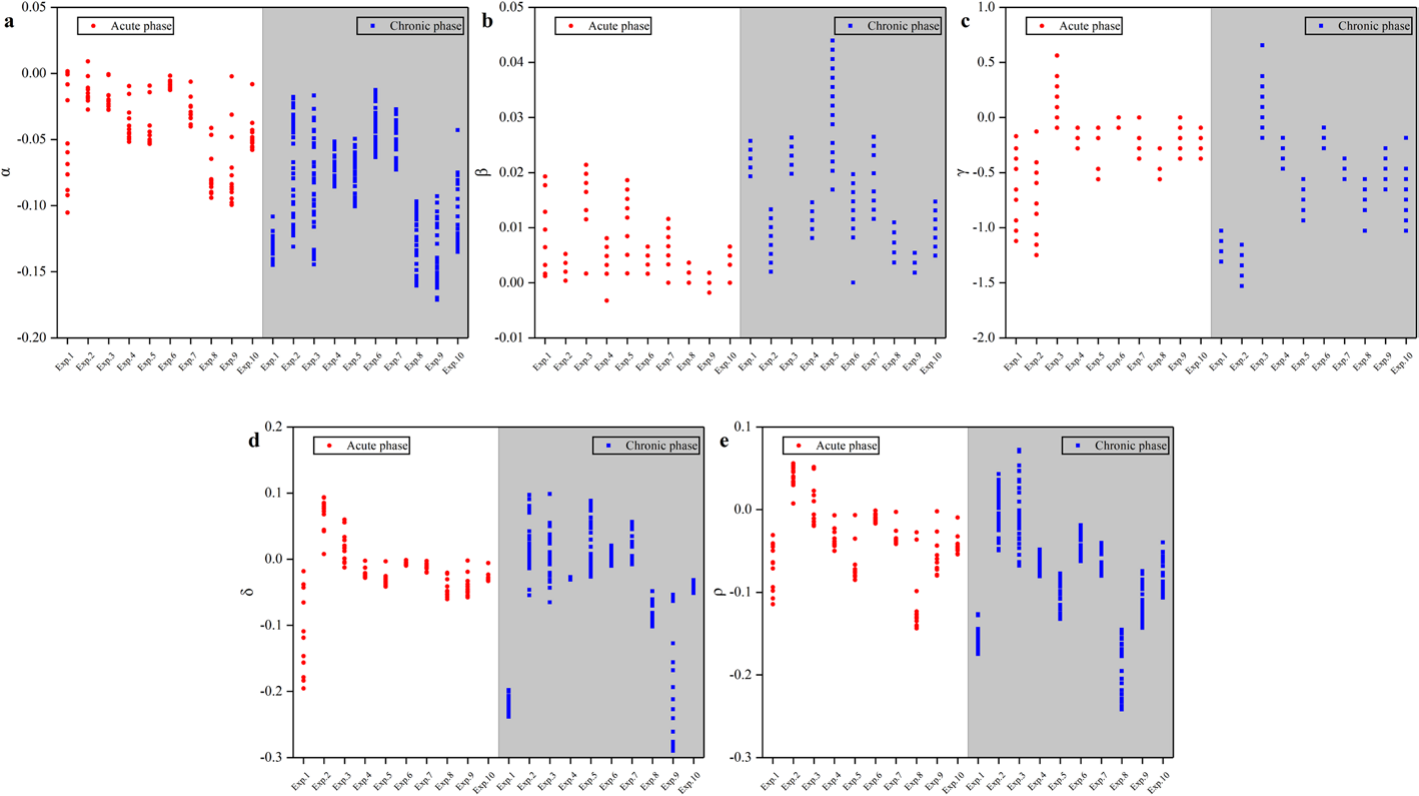
Characteristic parameters of cerebral edema rabbits within 0–6 h and within 6–24 h. Ten rabbits (Exp.1–Exp.10) in acute phase were listed with white background. Ten rabbits (Exp.1–Exp.10) in chronic phase were listed with gray background

Aiming at the distinction between the acute and chronic phases of cerebral edema, this study also evaluated those characteristic parameters data within 0–6 h and 6–24 h. We assumed that it is possible to distinguish between the acute and chronic phases of cerebral edema. Figure 6 plots the characteristic parameter of rabbits in the experimental group within 0–6 h and within 6–24 h, during which cytotoxicity and vasogenic cerebral edema, respectively, dominate the process in this cerebral edema model [2, 56]. The contrast between the acute phase and the chronic phase of rabbits is not as clear as that between experimental group and control group. Among these characteristic parameters, the distribution in acute/chronic phase also overlaps partly.

Table 3 lists the ROC data of 0–6 h vs 6–24 h in experimental group based on every possible combination of characteristic parameters. It turns out that AUC of $\alpha \beta \gamma \delta \rho$ was highest, reaching 0.9326.

**Table 3:** ROC results of 0–6 h vs 6–24 h distinction in experimental group based on every possible combination of characteristic parameters

Single		Double		Triple		Quadruple		Quintuple	
Characteristic parameter	AUC^*^	Characteristic parameter	AUC	Characteristic parameter	AUC	Characteristic parameter	AUC	Characteristic parameter	AUC
$\alpha$	0.83	$\alpha \beta$	0.88	$\alpha \beta \gamma$	0.88	$\alpha \beta \gamma \delta$	0.87	$\alpha \beta \gamma \delta \rho$	0.93
$\beta$	0.83	$\alpha \gamma$	0.81	$\alpha \beta \delta$	0.90	$\alpha \beta \gamma \rho$	0.91		
$\gamma$	0.65	$\alpha \delta$	0.88	$\alpha \beta \rho$	0.91	$\alpha \beta \delta \rho$	0.90		
$\delta$	0.64	$\alpha \rho$	0.88	$\alpha \gamma \delta$	0.89	$\alpha \gamma \delta \rho$	0.93		
$\rho$	0.69	$\beta \gamma$	0.83	$\alpha \gamma \rho$	0.88	$\beta \gamma \delta \rho$	0.91		
		$\beta \delta$	0.85	$\alpha \delta \rho$	0.88				
		$\beta \rho$	0.88	$\beta \gamma \delta$	0.84				
		$\gamma \delta$	0.83	$\beta \gamma \rho$	0.86				
		$\gamma \rho$	0.84	$\beta \delta \rho$	0.88				
		$\delta \rho$	0.89	$\gamma \delta \rho$	0.88				

*AUC takes two significant digits

**Fig. 7:**
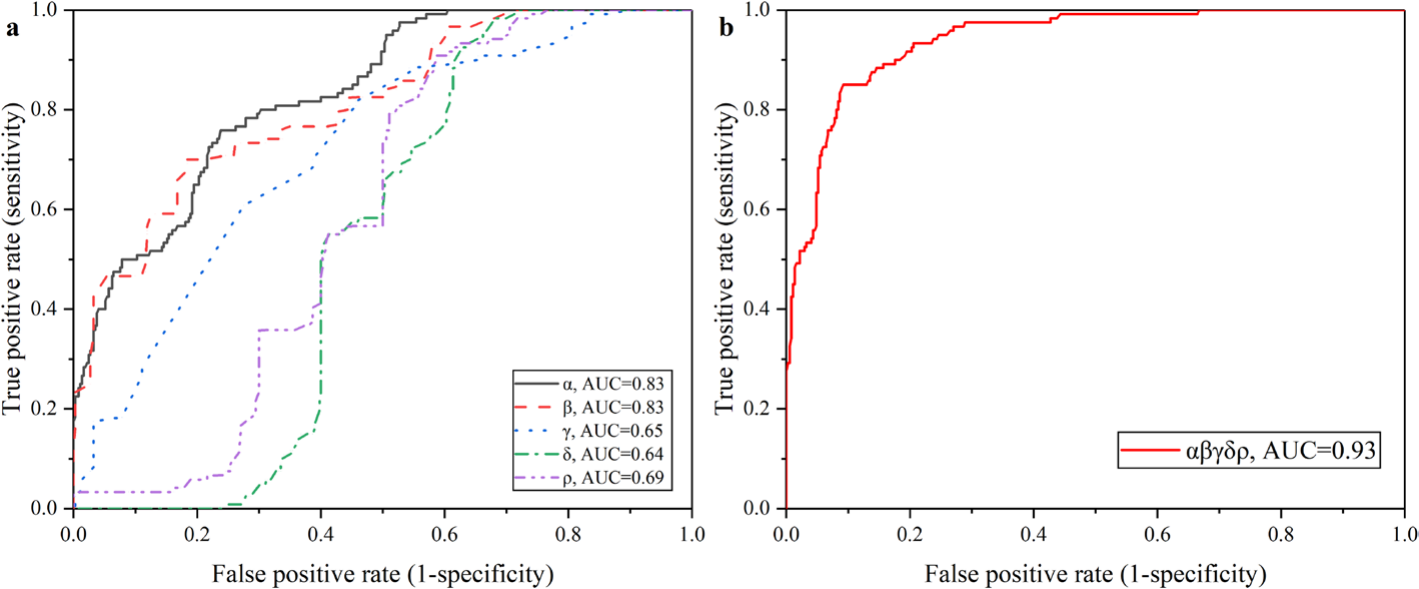
ROC curve of experimental group vs control group distinction within 1 h. **a** ROC curves based on single characteristic parameter; **b** ROC curves based on $\gamma \delta \rho$

Figure 7 plots the ROC curve of 0–6 h vs 6–24 h in experimental group. The AUC of single characteristic parameters were also relatively poor, of which all were lower than 0.85. When combined all these five characteristic parameters, the optimal discrimination index can be achieved, reaching 85.0% sensitivity and 91.0% specificity.

## Discussion

As a high-incidence non-specific pathological swelling, cerebral edema seriously affects the prognosis. Especially for those hospitalized patients suffering from stroke, monitoring is urgently required. Electromagnetic induction measurement has high application prospects in bedside and point-of-care monitoring. Recently, Alruwailli et al. showed that resonant skin patch sensors can be used to detect changes in the cardiac intraventricular stroke volume, thereby providing a non-invasive monitoring index for stroke patients [57]. Mohammed et al. proposed a wearable readout system to carry out hemodynamic detection by extracting the radio frequency attributes of resonant frequency shift and magnitude variation [58, 59]. This study demonstrates that, combined with our specific algorithms, the detection and distinction of acute/chronic cerebral edema can be realized in a non-invasive and bedside manner. The next iteration of development will be integration of hardware and algorithms into a wearable system to carry out clinical trials, through which may improve the prognosis.

This study focuses on the amplitude-frequency data within the 3 dB bandwidth near the characteristic frequency. Hui et al. pointed out that, for the electromagnetic detection signals, the amplitude data have high stability and poor sensitivity. In contrast, the phase data have high sensitivity and poor stability. The phase data are more vulnerable to the change in relative position of the measured object [60]. Therefore, we chose the amplitude data and managed to improve the sensitivity through reasonable processing and analysis. In addition, many studies believed that the S-parameters data at this frequency point are correlated with intracranial changes [20, 61, 62]. Studies by several teams such as Li, Pan, Oziel, and so on have focused on the data features near the characteristic frequency [42, 50, 55, 61, 63]. At this frequency, the two-port network indeed reached best impedance matching. Also, the radiation field radiated by the sensor has the strongest energy at this frequency. When the energy of this field reached largest, the weak disturbance of pathophysiological changes in biological tissues can modulate the near-electromagnetic field received by port 2 to the greatest extent, resulting in the maximum change of S-parameters in the frequency band.

Furthermore, five characteristic parameters were proposed in this study. Brain’s equivalent impedance $Z = R + j\omega X$ will change due to intracranial lesions. This disturbance caused by intracranial lesions will have a complex impact on the electrical parameters of this two-port network, which may change the sensor's self-inductance $L$, parasitic capacitance $C$, and parasitic resistance $R$. The shift of the characteristic frequency to higher frequency does not necessarily represent the decrease of $C$. It may also be caused by the change of $L$. Similarly, the improvement in gain may not only be related to the change of $R$. The Ab-CPE algorithm comprehensively considers the change characteristics of the amplitude-frequency data near the characteristic frequency and the coupling mechanism between brain and the two-port network. The frequency where meets the maximum coupling coefficient of the coil sensor and the coupling degree can be reflected by parameter $\alpha$ and $\beta$. The parameter $\gamma$ extracted by the 3 dB bandwidth combined the multi-frequency data to comprehensively reflect the change of $Z$. Then, the parameters $\delta$ and $\rho$ were a composite transform on the former parameters. The results showed that although the single parameter-based result is not optimal, the optimal combinations of those characteristic parameters in cerebral edema detection and phase discrimination both contained $\gamma$, $\delta$, and $\rho$. This showed that the availability of multi-frequency data is higher. Combining characteristic parameters at multiple frequencies can improve robustness and accurately reflect the difference between different intracranial conditions. This also explains the complexity of brain’s equivalent impedance change.

During the analysis of cerebral edema detection, the variations in S-parameters data were basically caused by metabolic activities such as blood supply and oxygen supply in control group. Compared with the drastic changes caused by brain edema, all the characteristic parameters of the control group were near 0. Among those parameter combinations for the detection of cerebral edema, $\gamma \delta \rho$ is the best. This is also due to the high discrimination of the experimental group and the control group. Triple combination was enough for distinction and more parameters’ combination may even bring unnecessary information. Evidently, the AUC results of quadruple and quintuple combinations showed that they are also capable for the detection requirements, and we just select the optimal solution.

During the analysis of acute/chronic phase distinction, the distinction performance rose with multiple parameters included, reaching the highest when all parameters were enrolled. This means that each parameter was slightly different in the acute and chronic phase. Incorporating multiple parameters can improve the distinction accuracy. The reason why the best characteristic parameter combination in the acute/chronic phase distinction and detection of cerebral edema was different may be caused by the algorithm procedures. In the Ab-CPE algorithm, combination of multiple parameters needs to be mapped to $\left[ { - 1,1} \right]$ for Euclidean distance calculation. The true value ranges of those parameters under the experimental design of detection and distinction are different. Thus, the relationship between different parameters may change after mapping, which leads to divergence in optimal solutions. This revealed that, in the subsequent clinical experiments, the electromagnetic induction data should be collected in a massive manner, by which we can obtain multiple characteristic parameters through specific algorithms. After that, iterative analysis and trial of all parameter combinations can be conducted to determine the optimal solution. The Ab-CPE algorithm proposed in this study provided a feasible solution for the clinical data in future researches.

Certainly, this study also has some limitations. This study currently only analyzed the transmission parameter $S_{21}$ in the S-parameter data. The next step may be the comprehensive analysis of all characteristics of this two-port test system by integrating all S-parameters. In addition, this study focused on the narrow frequency band near the low-frequency characteristic frequency. Considering that biotissues have different coupling mechanisms in low-, medium-, and high-frequency electromagnetic fields, it is also necessary to broaden the working frequency [51, 64]. In the next stage, we will consider methods such as adding switch matrix to carry out broadband research.

## Conclusion

The multi-frequency and multi-parameter electromagnetic induction measurement data have great potential in the detection and monitoring of cerebral edema. Aiming at improving the effectiveness of cerebral edema detection and monitoring, this research proposed an Amplitude-based Characteristic Parameter Extraction (Ab-CPE) algorithm for characteristic analysis. It has been demonstrated that multi-frequency measurement data, combined with proper algorithm, have advantages over the traditional measure design. Notably, it is worthwhile to extract multi-dimensional characteristic parameters based on both coupling mechanism and data features for evaluation, through which the suitable parameter combination can be selected to improve accuracy. This research laid the foundation for the next step of multi-parameter and multi-sensor array research.

## Methods and materials

### Principle of electromagnetic induction detection

**Fig. 8:**
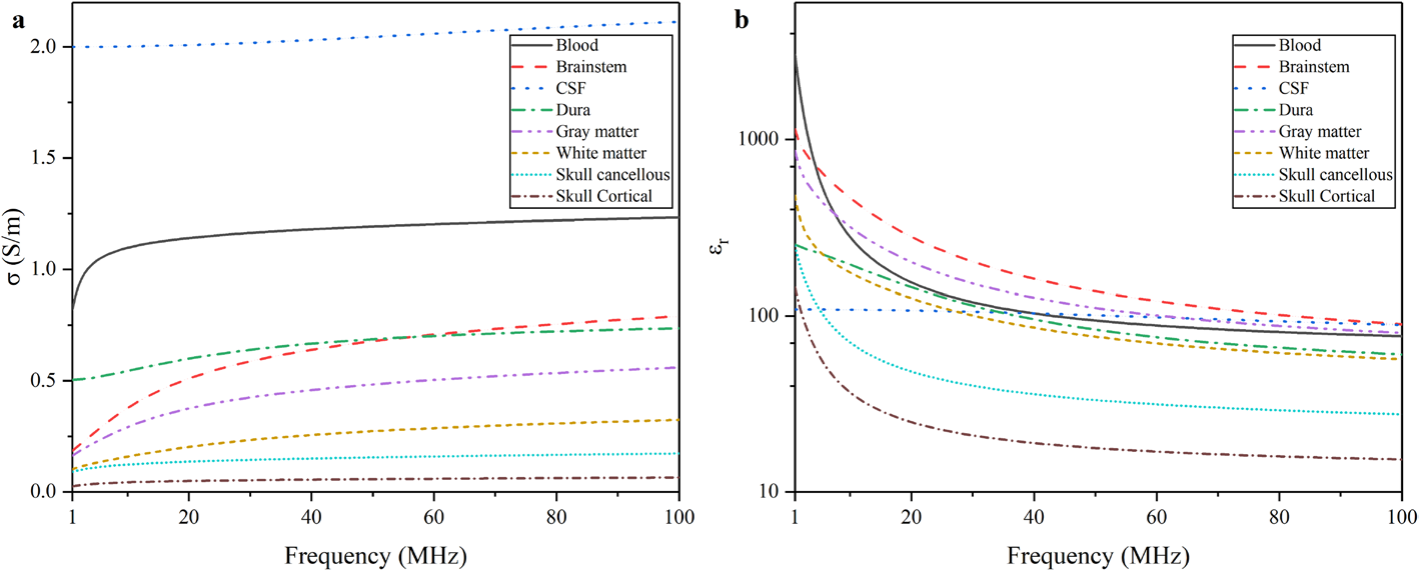
Dielectric properties of brain tissues in 1–100 MHz. **a** Conductivity $\sigma$; **b** relative permittivity $\varepsilon_{r}$

The dielectric properties of biotissues are frequency-dependent. Gabriel et al. measured the dielectric properties of various tissues over a wide frequency band. This database is widely used in electromagnetic induction-based detection studies [65–67]. Figure 8 shows the conductivity and relative permittivity of several brain tissues in 1–100 MHz. It can be found that the dielectric properties of brain tissues are frequency-dependent.

It can be found from Fig. 8 that the conductivity and permittivity of those brain tissues change with frequency, where different tissues have specific trend. For example, the $\sigma$ and $\varepsilon_{r}$ of the cerebrospinal fluid (CSF) do not significantly change in this frequency band and it has almost a linear relationship with frequency. By comparison, there is a non-linear relationship between blood’s dielectric properties and frequency. Based on this fact, when intracranial components change relatively due to cerebral edema, the average conductivity and relative permittivity of the brain will change [68]. Furthermore, at different frequencies, the change trend of the whole intracranial dielectric parameters may be different.

**Fig. 9:**
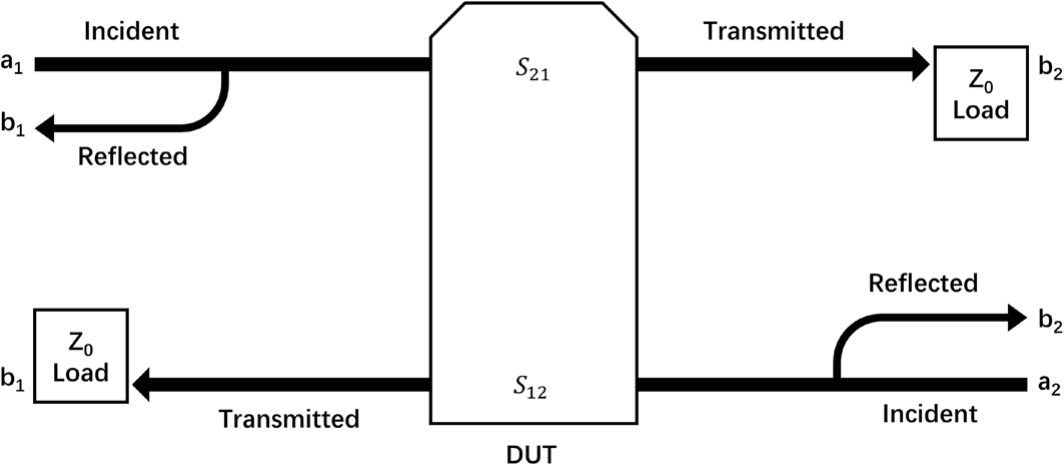
Principle of two-port network

According to the two-port network theory, bio tissue can be treated as the device under test (DUT). Figure 9 sketches out the principle of the two-port network system. At a specific frequency, the brain can be equivalent to a frequency-dependent complex impedance $Z_{L} = R + j\omega X$. This complex impedance $Z_{L}$ will show characteristic change when intracranial lesion occurs. Furthermore, this characteristic change can be extracted by measuring $S_{21}$ in scattering parameter (S-parameter) matrix

1 $$ \left[ {\begin{array}{*{20}l} {b_{1} } \\ {b_{2} } \\ \end{array} } \right] = \left[ {\begin{array}{*{20}l} {S_{11} } & {S_{12} } \\ {S_{21} } & {S_{22} } \\ \end{array} } \right]\left[ {\begin{array}{*{20}l} {a_{1} } \\ {a_{2} } \\ \end{array} } \right], $$

where $a_{1}$, $a_{2}$ are the incident wave and $b_{1}$, $b_{2}$ are the reflected wave of port 1 and port 2, respectively. Consequently, $S_{21}$ can be calculated by

2 $$ S_{21} = \frac{transmitted}{{incident}} = \left. {\frac{{b_{2} }}{{a_{1} }}} \right|_{{a_{2} = 0}} . $$

Studies have shown that because of the frequency dependence of the brain tissues’ dielectric parameters, the S-parameters in the two-port network will show different amplitude within a given measurement frequency range. When $S_{21}$ has maximum value, the coupling coefficient is highest between brain and the two-port network. At this frequency, the electromagnetic wave which radiated from the port 1 penetrates the brain tissue and transmits to port 2 to the greatest extent [61–63, 69]. Using vector network analyzer (VNA) to carry out sweep measurements, the measurement data at different frequencies can be obtained for characteristic analysis.

### Measurement system and data collection

**Fig. 10:**
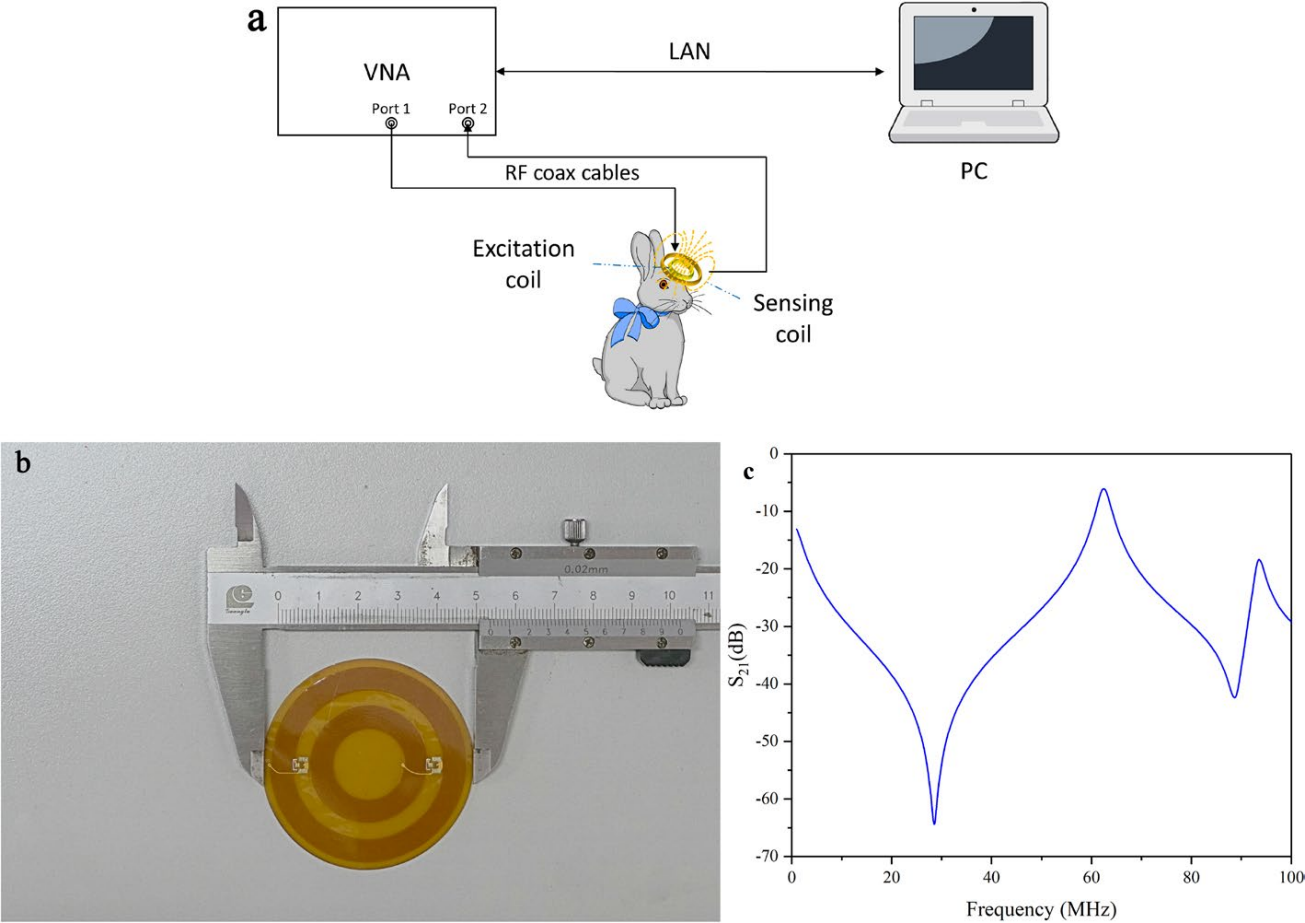
**a** Diagram of electromagnetic induction detection system; **b** flexible conformal electromagnetic sensor; **c** amplitude–frequency curve of sensor without measured object

Figure 10 shows the system diagram, the picture of the sensor, and its electromagnetic characteristics. The system consists of a RF vector network analyzer (Agilent E5061B, Keysight, USA), a homemade flexible conformal electromagnetic sensor which is desirable for local focusing measurement of CE [69], PC, and the Amplitude-based Characteristic Parameter Extraction algorithm. The VNA generates an excitation signal of a certain frequency, which is transmitted to the reference and port 1 through splitter. The excitation signal generates an electromagnetic field near the head via the sensor’s excitation coil. The receiving coil collects the transmission signal and transmits it to port 2 [61, 63, 69, 70]. The system parameters are set as follows. Sweep frequency range: 1–100 MHz; frequency points 1060; intermediate frequency (IF) bandwidth: 30 kHz; signal power: 10dBm.

In this study, 14 rabbits (available from Daping Hospital, 2.0–3.0 kg) were enrolled in the animal experiments. Rabbits were arrived 1 day before experiments and housed under ambient conditions (22 °C, 50% relative humidity, and a 12-h light/dark cycle), with free access to water and chow. The rabbits were randomly divided into experimental group ($n = 10$, marked as Exp.1, Exp.2, …, Exp.10) and control group ($n = 4$, marked as Con.1, Con.2, Con.3 and Con.4).

**Fig. 11:**
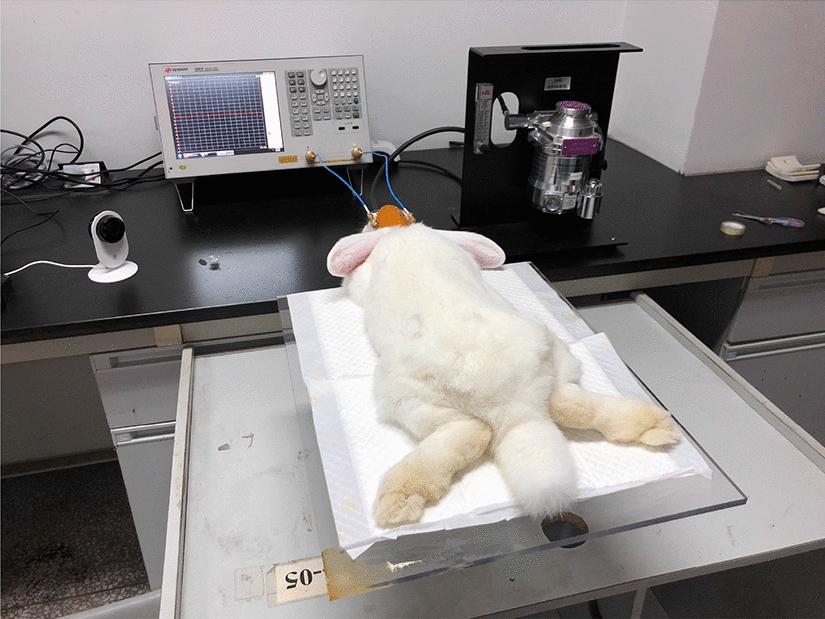
24-h real-time monitoring experiments in rabbits

Our previous studies had proved the validity of the cerebral edema model established by epidural liquid nitrogen freezing method [55, 69, 71]. Thus, this model was still utilized in experimental group. Also, in contrast, rabbits in control group experienced the same procedure but without freezing. After the establishment of cerebral edema and control model, rabbits were fit on the board and monitored for 24 h while the flexible conformal electromagnetic sensor was placed close to the freezing point of the head. The sensor was fixed on rabbit’s head by medical transparent adhesive tape to prevent body movement-induced relative displacement. This sensor can monitor local cerebral edema and has good bending robustness [69]. The measurement interval is set to once every 30 min. Rabbits in both groups were euthanized via IV pentobarbital overdose at the end of monitoring. Experimental arrangement is shown in Fig. 11.

### Amplitude-based characteristic parameter extraction algorithm

In this research, we proposed an Amplitude-based Characteristic Parameter Extraction (Ab-CPE) algorithm and input the measurement data for further characteristic analysis. Notably, this study selected the frequency points near characteristic frequency for further analysis. The Ab-CPE steps are as follows:

1) Define the measurement start time $t_{0}$ (the first measurement time). Record the amplitude-frequency curve of $S_{21}$ at $t_{0}$ and define $A_{0} \left( {f_{0} } \right) = {\text{max}}\left( {\left. {S_{21} } \right|_{{t_{0} }} } \right)$. $f_{0}$ is the frequency where $A_{0} \left( {f_{0} } \right)$ is. Then, find the lower frequency $f_{0}^{^{\prime}}$ and upper frequency $f_{0}^{^{\prime\prime}}$ where $S_{21} \left( {f_{0}^{^{\prime}} } \right) = S_{21} \left( {f_{0}^{^{\prime\prime}} } \right) = A_{0} \left( {f_{0} } \right) - 3, f_{0}^{^{\prime}} < f_{0} < f_{0}^{^{\prime\prime}}$. After, the 3 dB bandwidth (half-power bandwidths) $F_{0}$ at time $t_{0}$ is

3 $$ F_{0} = f_{0}^{^{\prime\prime}} - f_{0}^{^{\prime}} . $$

Denote $N_{0}$ as the number of frequency points contained in the 3 dB bandwidth. Furthermore, calculate the mean amplitude of all frequency points in $F_{0}$ at $t_{0}$

4 $$ \overline{a}_{0} = \mathop \sum \limits_{n = 1}^{N} S_{21} \left( {f_{n} } \right)/N. $$

2) Define the subsequent *i*th measurement as $t_{i}$. Record the amplitude–frequency curve of $S_{21}$ at $t_{i}$ and define $A_{i} \left( {f_{i} } \right) = {\text{max}}\left( {\left. {S_{21} } \right|_{{t_{i} }} } \right)$. $f_{i}$ is the frequency where $A_{i} \left( {f_{i} } \right)$ is. Then, find the lower frequency $f_{i}^{^{\prime}}$ and upper frequency $f_{i}^{^{\prime\prime}}$ where $S_{21} \left( {f_{i}^{^{\prime}} } \right) = S_{21} \left( {f_{i}^{^{\prime\prime}} } \right) = A_{i} \left( {f_{i} } \right) - 3, f_{i}^{^{\prime}} < f_{i} < f_{i}^{^{\prime\prime}}$. After, the 3 dB bandwidth $F_{i}$ at time $t_{i}$ is

5 $$ F_{i} = f_{i}^{^{\prime}} - f_{i}^{^{\prime\prime}} . $$

Denote $N_{i}$ as the number of frequency points contained in the 3 dB bandwidth. Define the mean amplitude of all frequency points in $F_{0}$ at $t_{i}$

6 $$ \overline{a}_{i} = \mathop \sum \limits_{n = 1}^{N} S_{21} \left( {f_{n} } \right)/N_{0} ,f_{n} \in F_{0} . $$

Define the 3 dB bandwidth followed mean amplitude of all frequency points in $F_{i}$ at $t_{i}$:

7 $$ \overline{{a_{i}^{^{\prime}} }} = \mathop \sum \limits_{n = 1}^{N} S_{21} \left( {f_{n} } \right)/N_{i} , f_{n} \in F_{i} . $$

3) Define maximum characteristic parameter $\alpha$:

8 $$ \alpha = \left[ {A_{i} (f_{i} } \right) - A_{0} (f_{0} )]/A_{0} (f_{0} ). $$

4) Define frequency shift characteristic parameter $\beta$:

9 $$ \beta = \left( {f_{i} - f_{0} } \right)/f_{0} . $$

5) Define 3 dB characteristic parameter $\gamma$:

10 $$ \gamma = \left( {F_{i} - F_{0} } \right)/F_{0} . $$

6) Define mean amplitude characteristic parameter $\delta$:

11 $$ \delta = \left( {\overline{a}_{i} - \overline{a}_{0} } \right)/\overline{a}_{0} . $$

7) Define followed mean amplitude characteristic parameter $\rho$:

12 $$ \rho = \left( {\overline{{a_{i}^{^{\prime}} }} - \overline{a}_{0} } \right)/\overline{a}_{0} . $$

### Characteristic analysis based on the Ab-CPE algorithm

**Fig. 12:**
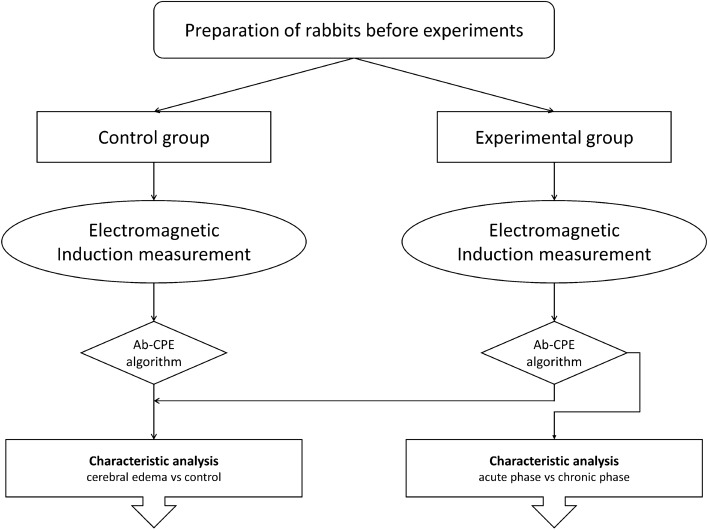
Flow diagram of Ab-CPE algorithm

Figure 12 shows the diagram of characteristic analysis based on the Ab-CPE algorithm. The data analysis in this study was conducted by MATLAB (MathWorks, Inc., USA), by which the Ab-CPE algorithm and ROC analysis was carried out.

We assumed that the characteristic parameters extracted from this Ab-CPE algorithm can be used to detect cerebral edema and distinguish the acute and chronic phase. For single characteristic parameter, threshold $T$ is set for binary classification. For the combination of multiple parameters, we use Euclidean distance to set the threshold $T$ and map these parameters to $\left[ { - 1,1} \right]$ to eliminate the influence of value difference between those characteristic parameters. Take parameter $\alpha$ and $\beta$ as an example

13 $$ \alpha_{i} \le T \le \alpha_{j} , i\left\{ {Exp.} \right\},j\left\{ {Con.} \right\} $$

14 $$ \sqrt {\left( {\frac{{\alpha_{i} }}{{\max \left( {\left| {\alpha_{i} } \right|} \right)}}} \right)^{2} + \left( {\frac{{\beta_{i} }}{{\max \left( {\left| {\beta_{i} } \right|} \right)}}} \right)^{2} } \le T \le \sqrt {\left(\frac{{\alpha_{j} }}{{\max\left( {\left| {\alpha_{j} } \right|} \right)}}\right)^{2} + \left(\frac{{\beta_{j} }}{{\max\left( {\left| {\beta_{j} } \right|} \right)}}\right)^{2} } , i\epsilon\left\{ {Exp.} \right\},j\epsilon \left\{ {Con.} \right\}, $$

where $\left\{ {Exp.} \right\}$ represents the $\alpha$ value of the experimental group within 24 h, and $\left\{ {Con.} \right\}$ represents the $\alpha$ value of the control group within 24 h. Here, we set $mean\left( {\left\{ {Con.} \right\}} \right) \ge mean\left( {\left\{ {Exp.} \right\}} \right)$ for expression purpose. Using Eq. (13), (14) for every possible combination of characteristic parameters, the performance of cerebral edema detection can be evaluated. Furthermore, take only those data where $\left\{ {Exp.} \right\}^{\prime} = \left\{ {\left. {Exp.} \right|0 - 1h} \right\}$ and $\left\{ {Con.} \right\}^{\prime} = \left\{ {\left. {Con.} \right|0 - 1h} \right\}$, we can evaluate the detection performance in early stage.

Similarly, different $T^{\prime}$ in Eqs. (15) and (16) can evaluate the capability of Ab-CPE in distinction between acute and chronic phase. Take parameter $\alpha$ and $\beta$ as an example

15 $$ \alpha_{i} \le T^{\prime} \le \alpha_{j} ,i\epsilon\left. {\{ Exp.} \right|0 - 6h\} ,j\epsilon\left. {\{ Exp.} \right|6 - 24h\} $$

16 $$  \sqrt {\left( {\frac{{\alpha_{i} }}{{\max \left( {\left| {\alpha_{i} } \right|} \right)}}} \right)^{2} + \left( {\frac{{\beta_{i} }}{{\max \left( {\left| {\beta_{i} } \right|} \right)}}} \right)^{2} } \le T^{\prime} \le \sqrt {\left( {\frac{{\alpha_{j} }}{{\max \left( {\left| {\alpha_{j} } \right|} \right)}}} \right)^{2} + \left( {\frac{{\beta_{j} }}{{\max \left( {\left| {\beta_{j} } \right|} \right)}}} \right)^{2} } ,i\epsilon\left. {\{ Exp.} \right|0 - 6h\} ,j\epsilon\left. {\{ Exp.} \right|6 - 24h\} . $$

## Data Availability

All data generated or analyzed during this study are included in this published article.

## Abbreviations

Ab-CPE: Amplitude-based characteristic parameter extraction
TBI: Traumatic brain injury
BBB: Blood–brain barrier
CT: Computed tomography
MRI: Magnetic resonance imaging
ICP: Intracranial pressure
GCS: Glasgow Coma Scale
TCD: Transcranial Doppler sonography
NIR: Near-infrared spectroscopy
EIT: Electrical Impedance Tomography
CCETI: Capacitively Coupled Electrical Impedance Tomography
MIPS: Magnetic induction phase shift
S-parameter: Scattering parameter
VNA: Vector network analyzer
IF: Intermediate frequency
ROC: Receiver-operating characteristic
AUC: Area under curve
